# Exosomes in the hypoxic TME: from release, uptake and biofunctions to clinical applications

**DOI:** 10.1186/s12943-021-01440-5

**Published:** 2022-01-17

**Authors:** Guangpeng He, Xueqiang Peng, Shibo Wei, Shuo Yang, Xinyu Li, Mingyao Huang, Shilei Tang, Hongyuan Jin, Jiaxing Liu, Sheng Zhang, Hongyu Zheng, Qing Fan, Jingang Liu, Liang Yang, Hangyu Li

**Affiliations:** Department of General Surgery, The Fourth Affiliated Hospital, China Medical University, Shenyang, 110032 China

## Abstract

Hypoxia is a remarkable trait of the tumor microenvironment (TME). When facing selective pressure, tumor cells show various adaptive characteristics, such as changes in the expression of cancer hallmarks (increased proliferation, suppressed apoptosis, immune evasion, and so on) and more frequent cell communication. Because of the adaptation of cancer cells to hypoxia, exploring the association between cell communication mediators and hypoxia has become increasingly important. Exosomes are important information carriers in cell-to-cell communication. Abundant evidence has proven that hypoxia effects in the TME are mediated by exosomes, with the occasional formation of feedback loops. In this review, we equally focus on the biogenesis and heterogeneity of cancer-derived exosomes and their functions under hypoxia and describe the known and potential mechanism ascribed to exosomes and hypoxia. Notably, we call attention to the size change of hypoxic cancer cell-derived exosomes, a characteristic long neglected, and propose some possible effects of this size change. Finally, jointly considering recent developments in the understanding of exosomes and tumors, we describe noteworthy problems in this field that urgently need to be solved for better research and clinical application.

## Introduction

Hypoxia (a low O_2_ level) is a hallmark of many solid tumor cells. Hypoxia develops in cancer because the distance between cancer cells and the vasculature exceeds oxygen diffusion limits (which are as great as ~ 200 μm, depending on the local oxygen concentration in blood plasma) [1, 2]; this failure mainly results from disordered neovasculature [3] or perfusion limitations caused by temporary obstruction [1, 2]. Although there is no clear threshold to distinguish normoxia from hypoxia in cancer [4], tumor tissues can be generally divided into three regions: the normoxic zone (with functional blood vessels nearby), hypoxic zone (~ 100 μm away from functional blood vessels [5], with a partial pressure of oxygen < 10 mmHg [3]), and necrotic zone (~ 150 μm away from blood vessels, with a very low oxygen concentration) [6]. Hypoxia has different effects throughout cancer progression; in particular, it can stimulate angiogenesis, ultimately leading to more malignant and lethal cancers. It has been established that cellular communication is more frequent and more complex in the hypoxic tumor microenvironment (TME), and accumulating evidence suggests that extracellular vesicles (EVs) participate in complex hypoxic processes by acting as signal transporters [7, 8].

EVs are defined as particles with a lipid bilayer membrane that contain components from donor cells but lack a functional nucleus; they are released by all cells and cannot replicate [9–11]. Although there are no doubts about the role of EVs in physiological and pathological processes, the fact that different types of EVs display overlapping biophysical properties but lack specific markers that can discriminate the subtypes perfectly are points of confusion for researchers, as is the nomenclature of EVs [12]. In the present review, we mainly focus on exosomes, MVB-derived EVs ranging from ~ 40 to 162 nm in diameter (~ 100 nm on average) [10], and present some features of other EV subtypes. The biogenesis of exosomes (of endosomal origin) involves double invagination and subsequent fusion of the plasma membrane. Generated by late-sorting endosomes (LSEs) evolving from early-sorting endosomes (ESEs), multivesicular bodies (MVBs) are the products of the first invagination; MVBs can release intraluminal vesicles (ILVs, pre-exosomes) as exosomes by merging with the plasma membrane after the second invagination or can fuse with lysosomes and autophagosomes and be degraded [10]. Similar to hypoxia, exosomes also are involved in various physiological and pathological processes, in which they act primarily as important messengers. As a result, there may be certain intrinsic relationships between hypoxia and exosomes in cancer, and these relationships are the topic of this review.

## Hypoxia can influence the release of exosomes

Cancer-associated cells secrete more exosomes than healthy cells due to the need for intercellular information or nutrient exchange [13]. As estimated, the number of exosomes contained in the blood of cancer patients is twofold greater than the number in healthy human blood [14], and tumoral granulocytic myeloid-derived suppressor cells (G-MDSCs) produce more exosomes than splenic G-MDSCs [15]. Therefore, it is reasonable to think that more exosomes are necessary to satisfy the cell communication needs in cancer because of the complicated environment of hypoxia that develops in tumors. This scenario has been proven in various cancers, including glioma [16], breast cancer [17, 18], hepatocellular carcinoma [19], pancreatic cancer [20], gastric cancer [21], colorectal cancer (CRC) [15, 22], and prostate cancer [23], and different functions are mediated by exosome cargoes. Interestingly, compared with hypoxia, hyperoxia can reduce the number of exosomes released in CRC [15]. Taken together, these results indicate that hypoxia exerts its effects on tumors by increasing the number of cancer cell exosomes, which can carry signals to recipient cells. Notably, hypoxia also induces an increase in the number of exosomes in noncancerous cells [24–26], which indicates that hypoxia universally induces increases in exosomes. However, the detailed mechanism by which hypoxia increases exosome release from cancer cells is still not well understood. Here, we will focus on potential but not exhaustive possibilities derived from published reports.

Cargo sorting, transport of MVBs and fusion with the plasma membrane are the key steps in exosome release, and hypoxia may influence these steps. Cargoes and cargo-sorting machinery are the first regulators of exosome release [27], and hypoxia may mediate their activity. Dual immunofluorescence analysis proved that RAB22A is enriched in the membranes of microvesicles (MVs), which indicates that RAB22A is an MV cargo, and under hypoxia, HIF-dependent overexpression of RAB22A was shown to be required for increased MV formation [28]. Although MVs are different than exosomes, that study provided some insights into hypoxic regulation of exosomal cargo, which affects exosomal release. Exosome markers, such as certain tetraspanin membrane proteins (CD81 and CD63) and TSG101, are also good indicators of hypoxic regulation. Some of these markers are both exosome cargoes and sorting mediators. CD63 is particularly enriched on the surface of exosomes and has been reported to function in endosomal sorting during melanogenesis [29]. The tetraspanins CD81, CD82 and CD9 are also directly involved in the sorting of various cargoes to exosomes [27]. Many researchers have demonstrated that tetraspanin is upregulated by hypoxia. For example, overexpressed CD63 and GLUT-1 are markers of hypoxia status and are associated with poor outcomes of GIST (gastrointestinal stromal tumors) patients [30]. These studies indirectly supported the idea that hypoxia exposure may affect cargo loading and the subsequent release of exosomes. Intracellular transport involves the association of organelles with the cytoskeleton (actin and microtubules) and associated molecular motors (dynein, kinesins and myosins) and molecular switches (small GTPases) [27]. The actin cytoskeleton [31], microtubules [32] and molecular motors [33] are thought to change in different cells under hypoxic conditions. A possible example is invadopodia, invasive actin structures and key secretion sites for exosomes [34]. Evidence has shown that hypoxia can promote the formation of invadopodia [35, 36]. Moreover, as mentioned above, MVBs fuse with lysosomes, leading to degradation, or with the plasma membrane, leading to the release of ILVs as exosomes. When one of these two pathways is blocked, MVBs will enter the other pathway. Although the regulation of the balance between the degradation and secretion of multivesicular endosomes (MVEs) remains largely unexplored, how hypoxia increases the release of exosomes in this system can be explained. ISGylation of the MVB protein TSG101 by ISG15 can promote lysosomal degradation to inhibit the release of exosomes [37]; in addition, as identified in another independent experiment, ISGylation is lower under hypoxic conditions [38]. It has also been demonstrated that RAB27b regulates the motility of MVEs toward the plasma membrane [39], both RAB27a and RAB27b facilitate the docking of MVEs to the membrane [39], RAB7 plays a role in transporting MVEs to lysosomes for degradation [40], and (coincidentally) hypoxia can increase exosome release by upregulating RAB27a and downregulating RAB7 in ovarian cancer cells [41]. These results indicate that hypoxia can participate in the intracellular transport of MVEs by blocking the degradation pathway, paving the way for MVE fusion with the plasma membrane and increasing exosome release in cancer; however, further verification is needed. The final step of exosome secretion, the fusion of MVEs with the plasma membrane, is mediated by SNARE proteins and synaptotagmin family members [42]. An increase in the SNARE protein SNAP-25 under hypoxia has been reported [43]. Pyruvate kinase type M2 (PKM2), whose expression is increased in hypoxia [44], can promote exosome secretion by phosphorylating synaptosome-associated protein 23 (SNAP-23) [45]. Interestingly, reduced exosome release has been observed in certain cases of neurobehavioral dysfunction [10], and hypoxia is known to be an enhancer of neurobehavioral dysfunction [46] (an effect that is opposite the effect in cancer, in which hypoxia promotes the release of exosomes to accelerate disease progression). Fei et al. observed that hypoxia can upregulate HSP70 expression and downregulate the expression of the presynaptic proteins syntaxin I, synaptic vesicle protein 2 (SV2) and synaptotagmin I (which are associated with EVs) to impair motor and sensory suppression functions [46]. However, it remains unknown whether hypoxia reduces exosome release by suppressing the expression of the presynaptic proteins syntaxin I, SV2 and synaptotagmin I to impair nerve functions, a hypothesis that is consistent with the protective role of exosomes in neurodegeneration [10]. If the hypothesis turns out to be true, the opposite conclusion regarding the mechanism in cancer can be reached, suggesting that hypoxia may promote exosome release by inducing the overexpression of synaptotagmin family members and associated factors to accelerate cancer progression. Moreover, low pH and an acidic microenvironment are clear results of hypoxia, and these characteristics facilitate exosome release and uptake [47]. This phenomenon implies that hypoxia can indirectly benefit the release and uptake of exosomes. Another indirect example is that hypoxia induces exosome release in a calcium-dependent manner through MCT1 and CD147 [48]. However, it remains to be seen which of the aforementioned direct and indirect effects of hypoxia is most important.

On the basis of this discussion, we conclude that hypoxia participates in the processes of exosome release and MVB degradation by influencing many key and auxiliary factors. However, how hypoxia influences or interacts with these key molecules remains largely unclear. Inhibition of translation initiation, elongation or termination, adaptive protein synthesis, extensive protein modification and metabolic reprogramming are the main hypoxia-related regulatory mechanisms, and these mechanisms have been reviewed extensively by Lee et al. [3]. Furthermore, biofunctions are manipulated by a network that is affected by factors related to the posttranscriptional processing of premRNA transcripts, including alternative splicing; stabilization or degradation of the mRNA product; regulation of mRNA translation; and posttranslational modification, stabilization, or degradation of the protein product [49]. At the transcription level, overexpression of the hypoxia master regulator HIF, a transcription factor, is consistently associated with an increase in exosome release in hypoxic cancer cells. For instance, the mRNA expression of PKM2 is increased by hypoxia in a HIF-1-dependent manner [44]. Moreover, the basic helix-loop-helix transcription factor BHLHE40 was shown to be induced by hypoxia, and BHLHE40 knockdown reduced the release of exosomes in breast cancer cells [50]. In terms of protein modification, in addition to ISGylation, as mentioned above, phosphorylation also influences the biogenesis of exosomes. A recent research article showed that the phosphatase Shp2 negatively controlled small EV (sEV) biogenesis by directly dephosphorylating tyrosine 46 of syntenin, and we believe that the EVs in this article are likely exosomes [51]. Shp2 can affect HIF in cancer [52], and the role of protein phosphatases in the hypoxic cancer environment has gained some attention [53]. However, it is still unknown whether hypoxia can induce protein phosphatases to affect exosome biogenesis. ncRNAs may be another potential mediator between hypoxia and exosome release. A good example is the lncRNA HOTAIR, which has the ability to promote exosome secretion in hepatocellular carcinoma [54] and is upregulated under hypoxic conditions in several cell lines [55].

## Hypoxia can elevate tumor-derived exosomal heterogeneity

The heterogeneity of the exosome population, which is composed of exosomes with different sizes, different cargoes inside and outside, different functional impacts on recipient cells and different cellular origins [10, 56], is a reflection of the cell state [57], making exosomes potentially desirable diagnostic and prognostic tools. For example, a recent study successfully evaluated drug potency by detecting drug occupancy using synthetic probes [58]. Exosomes released by different cells contain different cargoes and markers, and exosomes originating from the same cell line can carry distinct constituents [14]. Therefore, heterogeneity is not only a promising aspect of exosome applications but is also among the biggest obstacles to a better understanding of exosomes [59, 60]. Here, we focus on hypoxia, which is related to increased complexity of exosome action, and point out the temporal order of exosome biogenesis, cargo loading, release, transport, binding to target cells, uptake and final biofunction, specifically noting changes in exosome biofunctions resulting from exposure to hypoxia after transport, binding and uptake.

### Hypoxia changes the size of exosomes in TME

Exosome size varies significantly even in a single cell line [56], probably due to intrinsic uneven invagination of the limiting membrane during exosome biogenesis [10], and it has been reported that the size of exosomes is associated with certain diseases. For example, a smaller exosome size (< 112 nm) in the pulmonary vein has been associated with a shorter time to relapse and shorter overall survival among non-small-cell lung cancer (NSCLC) patients [61]. There are many problems hindering our understanding of exosome size. First, the fact that each exosome-sizing technique has a unique bias with regard to exosome size estimates needs to be overcome [56]. For example, the detection limit of nanoparticle tracking analysis (NTA), which is widely used for exosome size detection for biological particle applications, is approximately 70 nm [62], although new technologies have been developed. The processes of preparing exosomes can lead to exosome shrinkage, swelling or flattening, and these changes have a clear impact on true size analysis [56]. Moreover, it is not possible to acquire totally purified exosomes due to technical limitations, which also influences the accuracy of size data. For instance, EVs in the typical exosome size range include apoptotic bodies, MVs, VLDLs and chylomicrons, retroviruses and exomeres, and these structures cannot be effectively separated from exosomes by centrifugation because they share similar densities and membrane orientations [59, 63]. While these factors have led to an unclear understanding of exosome size, hypoxia adds to this ambiguity. Exposure to hypoxia tends to lead to the release of smaller exosomes. Many results from different experiments with various cancer cells or cancer-related cell lines, such as colon cancer cells [22, 64], prostate cancer cells [23], pancreatic cancer cells [20], and BMSCs [65], have proved this trend. Some studies have proven that the Rab protein can influence the size of exosomes. Inhibiting RAB27 with targeted shRNAs can reduce the release of exosomes, but this manipulation also results in a significant increase in the presence of smaller endosome-sized vesicles (50 nm), which implies that Rab proteins have the ability to change the size distribution of exosomes [66]. In addition, substance-conservation studies have shown that when the release frequency of exosomes increases, the consumption of the membrane also increases. Under hypoxia, the membrane supply may not meet the membrane consumption requirements for exosome release, leading to the release of smaller exosomes. Although these studies have proven that hypoxia changes exosome size, many researchers have overlooked the subtle differences between cell lines and have concluded that normoxic and hypoxic cancer-derived exosomes are among the size range of exosomes [16, 18, 67]. In view of the common effect of hypoxia on exosome size and the contradictory conclusions obtained from studies, we discuss exosome size in the present review.

In 1989, Stephen Paget proposed the “seed-and-soil” hypothesis [68]. However, the “*soil-dandelion-soil*” version of this hypothesis seems to be a more reasonable metaphor for explaining the occurrence of cancer and metastasis. 1) Before primary cancer occurs, the *primary soil (pretumor site)* is made suitable for *dandelion (primary tumor cell*) survival through a multistep development process (which effectively explains the age dependency of cancer) [69, 70] under the influence of various factors, including the accumulation of mutations in somatic cells [70, 71], metabolic reprogramming [72, 73], microbiota changes [74], inflammation [72, 73], and obesity [72, 75]. Then, *dandelions (primary tumor cells*) grow uncontrollably and reshape the *primary soil*. 2) As tumors grow larger and metastasize, the *dandelion seeds (exosomes)* are released into the bloodstream and deposited in *distant soil (metastatic sites)* to form premetastatic niches (PMNs) for secondary tumor growth before circulating tumor cells arrive at these distant sites [76]; this idea has been proven through some independent experiments [77–80]. In two recent studies, hypoxia-induced exosomes from CRC cells promoted liver-tropic metastasis by making *distant soil* fertile for the formation of PMNs [81], and hypoxic exosomes (HypoExos) from prostate cancer cells were found to upregulate the levels of matrix metalloproteinases (MMP2 and MMP9) and extracellular matrix proteins (fibronectin and collagen) and increase the number of CD11b + cells to enable PMN formation at selective sites [82]. In consideration of this explanation, it is reasonable to presume that smaller exosomes may be more easily transmitted via the bloodstream to metastatic sites to form a PMN when hypoxia changes the hemodynamics in cancer. In addition, smaller exosomes can cross physiological gaps easily to reach additional cells. When the total weight and density are the same, a smaller value means that more cells may be exposed to and affected by exosomes, triggering signaling cascades and ensuring the transmission of valid bioinformation. Moreover, smaller exosomes may be internalized faster than larger exosomes [83], indirectly indicating that smaller exosomes from hypoxic cancer environments may contribute to tumor progression more easily and more effectively. Another recent study provides an indirect relationship between the size of sEVs and tumor malignancy. Stiffness and osmotic pressure are positively correlated with EV malignancy and negatively correlated with the size of sEVs, while bending modulus is negatively correlated with EV malignancy and positively correlated with the size of sEVs [84]. Another study showed that exosomes derived from malignant ascites have a wider variation in size than those from nonmalignant ascites [85], and a smaller size of exosomes from malignant ascites was not reported, which may have been a result of the detection limit of NTA. Fortunately, another clinical trial (NCT02310451) may provide useful information about exosome size as a biomarker in the future (Table 1). In addition, the following details are unclear: the extent to which small exosomes contribute to cancer progression; whether smaller exosomes from different kinds of cancer cells are absorbed faster; and the biological, physical and chemical factors that influence exosome size and the extent to which exosome size is affected by these factors. We believe that these important questions can be resolved with the development of methods for exploring exosome evolution.

**Table 1:** Clinical trials about exosome biomarkers

Investigators or contacts	Start time	Tumour	Estimated or actual enrollment	Time perspective	Origin	Potential marker	NCT number
Yuhui Shen et al. [86]	2017	Osteosarcoma	40	Prospective	Blood	RNA	NCT03108677
Shonit Punwani et al. [87]	2015	Prostate Cancer	130	Prospective	Blood	HER	NCT02935816
Shu Zhang et al. [88]	2018	Pancreatic Cancer	30	Prospective	Blood	mRNA	NCT03821909
Hyun Koo et al. [89]	2020	Lung Cancer	470	Retrospective	Blood	Protein	NCT04529915
Olivier Bouché et al. [90]	2021	Colorectal Cancer	80	Cross-Sectional	Blood	Macromolecules, integrins, metallo proteases	NCT04394572
Lei Li et al. [91]	2018	Ovarian Cancer	160	Prospective	Blood	miRNA, lncRNA	NCT03738319
Alice HERVIEU et al [92].	2018	Sarcoma	30	Prospective	Blood	Concentration	NCT03800121
Lin Miao et al. [93]	2017	Cholangiocarcinoma	80	Prospective	Blood	ncRNAs	NCT03102268
Henri MONTAUDIE et al. [94]	2014	Melanoma	15	Prospective	Blood	Concentration, size	NCT02310451
Julie ABRAHAM et al. [95]	2019	Non-Hodgkin B-cell Lymphomas	90	Prospective	Blood	CD20, PDL-1	NCT03985696
Roger Tun et al. [96]	2014	Prostate Cancer	2000	Prospective	Urine	RNA gene signature	NCT02702856
Carl A Olsson et al. [97]	2020	Bladder Cancer	3000	Prospective	Urine	the expression profiles of the sncRNAs	NCT04155359
CHIH-YUAN WANG et al. [98]	2016	Thyroid Cancer	22	Prospective	Urine	Uncertain	NCT02862470
Nicolas MOTTET et al. [99]	2020	Clear Cell Renal Cell Carcinoma	100	Prospective	Urine	CD9+/CA9+ exosomes, CD9+/VGEFR2+ exosomes, CD9+/CD63+/CD81+/CA9+ exosomes,	NCT04053855
Roger Tun et al. [100]	2016	Prostate Cancer	532	Prospective	Urine	3-gene expression	NCT03031418
Andrew Cowan et al. [101]	2015	Oropharyngeal Squamous Cell Carcinoma	30	Prospective	Primary cell cultures	Protein Signature	NCT02147418

### Hypoxia influences cargo-sorting mechanisms in cancer exosomes

Exosome cargoes (proteins, nucleic acids, glycoconjugates and lipids) and their corresponding functions have been investigated at length. Similar to exosome size, exosome cargoes exhibit heterogeneity, vary greatly in different cancer-derived exosomes and are enriched by hypoxia. In the following section, we discuss three main cargoes (proteins, glycoconjugates and lipids), as well as nucleic acids, to show how hypoxia changes these exosome cargoes.

#### Proteins

In terms of heterogeneity of exosome proteins, more than 3000 common proteins and more than 1000 unique proteins were reported to be detected in three exosome samples released by a single cell line [102]. Exosome proteins are multifarious and include integral exosomal membrane proteins, lipid-anchored outer membrane proteins, peripheral surface proteins, lipid-anchored inner membrane proteins, inner peripheral membrane proteins, exosomal enzymes, soluble proteins and bulk inclusions [56]. Based on the fact that exosomes are generated at both plasma and endosome membranes, limited carrying capacity and steric exclusion are the initial causes of heterogeneity. Differential protein distribution, gene expression and environmental factors add to this diversity [56]. We believe that selective loading is another nonnegligible cause of heterogeneity because the exosome protein content is not always in line with the ratio of cellular proteins [103, 104].

In one study, there were 130 upregulated and 129 downregulated exosomal proteins in cells from the hypoxic NSCLC A549 cell group compared to the normoxia group [105], showing that hypoxia enhances exosome heterogeneity. Interestingly, compared with exosomes from cells expressing ANGPLT4 (exosome-derived protein of angiopoietin-like 4), exosomes from ANGPLT4-knockdown cells induced significantly decreased A549 cell migration in the presence of different oxygen levels [105], indicating that hypoxia affects biofunctions at least partially through exosome-loaded proteins. However, the migration abilities of A549 cells treated with exosomes from hypoxic ANGPLT4-knockdown cells were still higher than those of cells treated with exosomes under normoxia [105], which demonstrated that exosome cargoes may function similarly and complement each other to perform their biological functions. Exosomes from hypoxic prostate cancer cells showed a greater percentage of plasma membrane- and nucleus-derived proteins, and a relatively low percentage of these proteins were derived from the extracellular space or cytoplasm [82]. G-MDSCs promote the stemness of CRC cells through exosomal S100A9, and hypoxia can promote exosome production in G-MDSCs in a HIF1α-dependent manner [15]. Proteins in tumor exosomes also participate in hypoxia-associated responses. For instance, breast cancer cell exosomes containing metastasis-associated protein 1 can be transferred to other cells to regulate the response to hypoxia [106]. Given this evidence and that of other studies not cited in this review, we suggest that hypoxia promotes many malignant phenotypes of cancer cells by changing exosome protein heterogeneity and that proteins in cancer-derived exosomes sometimes contribute to the hypoxia response.

The precise mechanisms critical for increased protein heterogeneity in HypoExos have been slowly determined partially because the exosomal protein-sorting mechanism remains obscure. Some potential possibilities based on the findings of recent related studies can be summarized. First, the simplest possibility is that more proteins in cancer cells indicate more proteins in exosomes. In a given cell type, when particular proteins increase in abundance, they occupy more space; therefore, it is presumed that more proteins will be included during exosome formation. Likewise, exosomes with fewer proteins are derived from cells with decreased protein expression. Hypoxia can promote protein synthesis by stimulating the formation of a complex that includes HIF-2α, RNA-binding motif protein 4 (RBM4) and eIF4E2 (an eIF4E homolog) that assembles at reverse hypoxia response elements (rHREs) and the formation of a hypoxia-specific eIF4F complex that binds rHREs to facilitate translation initiation or inhibit some protein production through various mechanisms, such as by suppressing translation [3]. However, the presence of more proteins in cells does not always indicate that there are more exosomal proteins because protein-sorting mechanisms may affect the number of exosomal proteins; as such, hypoxia may influence the key molecules associated with protein loading to influence the number and type of exosomal proteins. In addition to the tetraspanin family mentioned above, syntenin [27] and glycan signatures [107–109] are also involved in cargo sorting and are potential mediators of hypoxic effects. An early study showed that syntenin induces IGFBP-2 expression via HIF-1a activation to promote angiogenesis [110], indicating an association between hypoxia and syntenin. However, the details remain unexplored. Posttranslational modifications (PTMs) of proteins also affect the loading of specific elements into the ILVs of MVBs [104, 111]. Ubiquitination is the best example of a PTM that affects cargo loading. Ubiquitinated proteins can be recognized and made to accumulate via ubiquitin-binding domains in ESCRT-0 and ESCRT-II within microdomains of MVEs, limiting the amount of membrane available for exosome formation [27, 104, 112]. It has been widely reported that hypoxia can influence protein ubiquitination and ubiquitination-associated enzymes [113–115]. Although this finding remains unproven, it strongly implies that hypoxia can affect the protein-loading process of cancer cell exosomes by affecting ubiquitination. In addition to these features, the inner exosome membrane is enriched in molecular chaperones, which bind to aggregated and misfolded proteins [56], and hypoxia can result in the accumulation of misfolded proteins [3].

#### Nucleic acids

Currently, most researchers believe that exosomes contain various nucleic acids, including RNAs (mRNAs and ncRNAs, including lncRNAs, snRNAs, miRNAs, tRNAs, Y RNAs, vault RNAs, repetitive element RNAs and fragmented RNAs) and DNA sequences (DNA, single-stranded DNA, double-stranded DNA, genomic DNA, mitochondrial DNA and reverse-transcribed complementary DNAs) [56]. However, in a recent study, high-resolution density gradient fractionation and direct immunoaffinity capture were used to reassess exosome composition, and the findings showed that active secretion of cytoplasmic DNA and histones occurs through an autophagy- and MVE-dependent but exosome-independent mechanism, and exosomal RBPs (Ago1–4, RPS3, RPS8, EEF2, EEF1A1, hnRNPA2B1, PARK7/DJ1, GAPDH, and MVP) were found to be absent from classical exosomes [103]. These findings reflect the complexities of exosomes and the and insufficient knowledge surrounding the topic. Putting these unanswered questions aside, exosomal nucleic acids regulate the biology of tumors and are influenced by hypoxia. Few articles have reported an influence of hypoxia on cancer cell-derived exosome DNA. However, it has been widely reported that hypoxia impacts exosomal RNAs in cancer cells. For instance, hypoxia upregulated exosomal circ-133 to promote CRC metastasis [116] and increased the expression of miR-301a-3p to promote gastric cancer progression, metastasis, and EMT [21]. More evidence is available online and has been summarized by Wang and Kumar et al. [8, 117, 118].

Some articles provide some clues about how hypoxia changes exosomal RNA. First, from the aspect of cargoes, increasing evidence has proven that hypoxia regulates the expression of different ncRNA classes, and in some cases, these ncRNAs can influence HIF expression and stability, forming positive and negative feedback loops [119, 120]. miR-301a-3p is a good example. In one study, miR-301a-3p was upregulated both in hypoxic gastric cancer cells and exosomes released by these cells, and it was found to increase HIF-1α stability by targeting PHD3, forming a miR-301a-3p/PHD3/HIF-1α positive feedback loop [21]. A network underlying how hypoxia functions in RNA biogenesis is complex and involves many molecules. Here, we consider it in its simplest form, and the details are available in other reviews [120, 121]. HIF-1α and/or HIF-2α can directly target RNA elements (such as the HRE in the miR-155 promoter [122, 123]). Other HIF-independent factors include the AKT signaling pathway involving miR-21 [124]; conserved sites for the transcription factor Oct-4 in miR-210 [125], the transcription factor TWIST1 in miR-10b [126], and the transcription factor C/EBP-a/RUNX-1 in miR-424 [127]; sites for TET2 and TET3 (DNA-demethylating enzymes) in WT1 lncRNA [128], acetylation levels in the lncRNA-LET promoter region [129] and epidermal growth factor receptor (EGFR) suppression of some specific tumor-suppressor-like miRNAs in response to hypoxic stress through phosphorylation of argonaute 2 (AGO2) at Tyr 393 [130]. Drosha and Dicer, key enzymes involved in miRNA biogenesis, are downregulated under hypoxic conditions, as mediated by the ETS1/ELK1 transcription factors [131]. Of note, greater cargo production results in greater loading of exosomes. However, the regulatory mechanisms have been poorly elucidated to date, and other aspects should also be considered. miRNAs have been shown to be differentially sorted into exosomes according to their specific sequence (i.e., which may include specific motifs) [132], and hypoxia also affects RNA alternative splicing [133, 134] and RNA editing [135]. These effects may make specific RNAs more suitable for loading in cancer cell-derived exosomes.

In addition to affecting cargo-related factors, hypoxia may also influence “tools” used by cancer cells to load nucleic acids into exosomes, including RNA-binding proteins (RBPs, such as hnRNPA2B1 binding to the RNA GGAG motif, SYNCRIP directly binding to specific miRNAs enriched in exosomes sharing a common extra-seed sequence hEXO motif [136], Gags and Gag-like proteins [137] binding to another exosomal RNA sequence motif and the ESCRT-II subcomplex functioning as an RNA-binding complex), tetraspanin­enriched microdomains sequestering RNA­binding proteins in membrane subdomains or the miRISC, and protein AGO2 mediating RNA silencing processes and the KRAS–MEK signaling pathway (which acts through AGO2, major vault protein and Y-box-binding protein 1 (also known as YBX1)) [27, 56]. It has been proven that hypoxia has an effect on factors including hnRNP A2 (through pVHL, another important regulator of hypoxia [138]), tetraspanin, AGO2 [130], the KRAS-MEK signaling pathways [139–142], major vault protein [143, 144] and YBX1 [145]. Unfortunately, a small number of articles have studied hypoxia and these factors: YBX1 was found to mediate the selective loading of miR-133 and hnRNPA1 to mediate the selective loading of miR-1246 into HypoExos [145, 146], and other studies did not consider hypoxia. Therefore, to gain a better understanding and develop therapies for cancer, the effect of hypoxia urgently needs to be explored. Interestingly, as we emphasize above, exosome cargoes are not limited to contents in exosomes and include material carried on the outer surface of exosomes. Xu et al. found that a large number of exosomal miRNA species bound to RBPs reside on the outer surface of exosomes [147]. This finding is worth exploring more.

#### Glycoconjugates and lipids

Three main metabolic pathways (glycometabolism, lipometabolism and proteometabolism) and nucleotide metabolism are essential for mammals and are reprogrammed in hypoxic cancer cells. Even under normoxia, cancer cells undergo reprogramming of glucose metabolism as the tricarboxylic acid cycle is replaced by aerobic glycolysis, which is known as the “Warburg effect” [148]. Exosomes have the same topology as the cell [56]. Heterogeneous glycoconjugates containing different modules exist on the membrane of exosomes. Unfortunately, there are few reports about how hypoxia impacts exosome glycoconjugates. However, the analogy between exosomes and cells suggests that hypoxia may influence exosome glycoconjugates in cancer cells. First, cell membrane glycoconjugates play important well-characterized roles in cell-to-cell and cell−environment communications, and exosomes are crucial cell-to-cell messengers. Second, the glycoconjugate signature of cancer cell-derived exosomes is different from that of healthy cells [56], which means that exosome glycoconjugates are heterogeneous and changeable. Third, glycoprotein expression on the cell membrane can be affected by hypoxia [149]. In addition, glycans have the aforementioned roles in protein sorting and uptake, and hypoxic cells take up more exosomes in a proteoglycan-dependent manner [150]. Considering these notions, we believe that glycoconjugates in cancer cell-derived exosomes function as identifiable markers in bioinformation transmission and may be changed by the hypoxic TME to enable better exosome recognition or cargo sorting. Additional information about these possibilities is eagerly awaited.

The exosome membrane contains phosphatidylcholine (PC), phosphatidylserine (PS), phosphatidylethanolamine (PE), phosphatidylinositol (PI), phosphatidic acid (PA), cholesterol, ceramide, sphingomyelin, glycosphingolipids, and a number of lipids in lower abundance [56]. Some people have argued that PE and PS appear to participate in exosome biogenesis [56]. Neutral type II sphingomyelinase can hydrolyze sphingomyelin to produce ceramide [151], which directly promotes the budding of ILVs through its cone-like structure. In addition, ceramide can be metabolized to sphingosine 1-phosphate (S1P), which binds with inhibitory G protein (Gi)-coupled S1P receptors to promote exosome biogenesis [152]. Ceramide levels are increased by hypoxia and thus mediate various biological processes [153]. Whether hypoxia increases exosome release in this way is unknown. Depletion of ABCG1, a cholesterol lipid efflux pump, triggers tumor regression with the accumulation of EVs and their derivatives and cargoes [154]. PS in MVs isolated from hypoxia-induced stem cells plays a critical role in uptake by human umbilical cord endothelial cells (HUVECs) [155]. Triglyceride accumulation in prostate cancer cells and EVs induced by hypoxia supports growth and invasiveness following reoxygenation [156]. Additionally, the role of lipids in processes related to cell communication, such as transport across the plasma membrane cannot be ignored. According to recent research, distinct lipid compositions cause exosome uptake by homologous cancer cells [157]. Unfortunately, hypoxic effects on the function of exosomal lipids are not well established. However, some investigators have realized the importance of changes in exosome lipids induced by hypoxia [8, 158].

In summary, before loading, cargoes must be produced; subsequently, cargoes are marked, identified and transported along with the cytoskeleton to special intercellular sites, where many key molecules facilitate their internalization into MVBs, and these cargoes ultimately reside in the exosomes released by MVBs. Although details of the hypoxic effects in various cancer cells differ or remain elusive, hypoxia influences the amount, kind and state of cargo, and through sorting mechanisms, hypoxia can affect cargo loading in cancer cells. Exploring the concrete mechanisms of exosomal cargo loading in hypoxic cancer cells will greatly facilitate the development of exosomes that can be used to target the hypoxic TME for better cancer treatment.

## Extracellular transport, binding and uptake of exosomes under hypoxia

Here, exosome transport refers to the intermediate processes between release and uptake. Studies on exosome transport are relatively rare. Many researchers have reported that hypoxia can increase the number of exosomes and that most of these exosomes are transmitted in the blood. No studies have explored whether the transport of exosomes released from cells under different oxygen concentrations differs. However, at least thus far, the idea that changes in exosome size affect transport, as mentioned above, is plausible, but the details remain largely unexplored. As an indirect but useful example, an acidic environment is most suitable for exosome existence and isolation [159]. This notion indicates that short-distance exosome transport may benefit from a hypoxic and acidic microenvironment.

Hypoxia-derived exosomes exhibit higher uptake efficiency [18, 157], and hypoxic tumor cells take up more exosomes [150]. The principles of uptake are shared by exosomes and other subpopulations of EVs, and EV uptake is analogous to well-characterized models of virus–cell interactions [27, 56].

When EVs are transported to target cells, molecules on the surface of exosomes interact with the membrane of target cells. In this way, changes on both the surface of cells and exosomes influence recognition. These molecules include tetraspanins (for example, CD9, CD63 and CD81 [160]), CD44 [161], integrins [77], lipids, lectins (for example, CD171/Siglec-1 [162]), extracellular matrix (ECM) components (for example, fibronectin [163]), heparan sulfate proteoglycans (HSPGs; for example, Glypican-1 [164]), and intercellular adhesion molecules (ICAMs; for example, CD54 [160]). Details are available in corresponding references or the review by Laura Ann Mulcahy or van Niel et al. [27, 165]. The effects of hypoxia on tetraspanins and lipids are also discussed above. Hypoxia promotes recycling of the α6β4 (but not the α3β1) integrin through control of microtubule-dependent trafficking of RAB11-containing vesicles, which are considered EVs in MDA-MB-231 breast cancer cells [166], and exosomal integrin α6β4 is associated with lung metastasis through absorption by responding cells [77], which implies that hypoxia may influence the binding between exosomes and recipient cells by targeting integrin α6β4. In addition, integrins facilitate CD44 [161], ICAM [160], ECM [163] and tetraspanin [160] participation in the interactions between exosomes and target cells, and all these interactions are tightly associated with hypoxia (see the details in the following references: ICAM-1[167, 168], ECM [169], CD44 [170, 171], and tetraspanins [172]). HSPGs present in EVs and at the plasma membrane contribute to the docking and/or attachment of vesicles to recipient cancer cells [164]. A recent study showed that hypoxic glioma cells take up more exosome-like EVs in an HSPG-dependent manner than normoxic glioma cells [150]. Coincidentally, hypoxia can transcriptionally deregulate endosulfatase 1 [173], which removes sulfate moieties at the 6-O positions of glucosamine [174]; this finding is consistent with the trend of more exosomes being absorbed by cancer cells under hypoxia. An excellent recently published example showed that hypoxia- and low-pH-stimulated exosomes exhibited higher uptake efficiency [157]. Further experiments proved that low pH changes the lipid composition in the exosome membrane to enhance exosome uptake by homologous tumor cells [157]. Regretfully, although hypoxia also causes similar effects, it has not been further researched. Therefore, whether hypoxia directly functions at low pH by changing the exosome membrane composition or whether hypoxia indirectly forms an acidic microenvironment to enhance exosome uptake remains unclear.

**Fig. 1:**
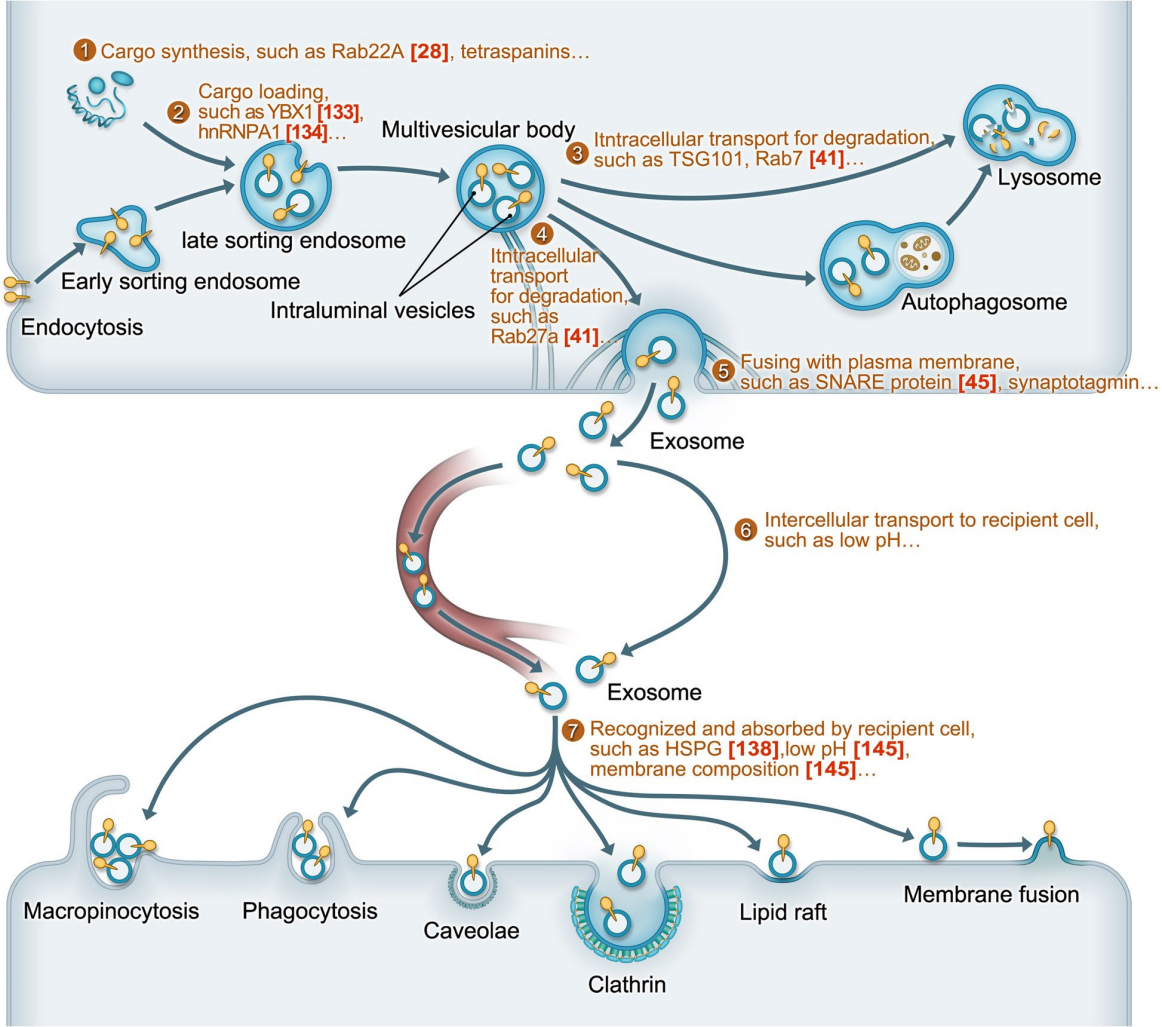
A schematic representation for the biological process of exosomes and key steps affected by hypoxia. ①Hypoxia affects cargo synthesis at translation level. ②Hypoxia influence exosome release and cargo-sorting by cargo-loading key tools (such as YBX1 and hnRNPA1). ③Hypoxia blocks MVBs degradation to increase exosome release (such as downregulating Rab7). ④Hypoxia causes overexpression of key molecules involving in MVBs transport towards plasma membrane to increase exosome release (such as Rab27). ⑤Hypoxia may increase exosome release by promoting the fusion between MVBs and plasma membrane (such as SNARE protein). ⑥Intercellular transport includes long-distance transport to distant recipient cells by circulation system (left) short-distance transport in local TME (right). Smaller exosome size induced by hypoxia seems to facilitate circulation transport, but it is not been proved. Acidic microenvironment, a result of hypoxia, may be beneficial to transport under local acidic microenvironment. ⑦Hypoxia and low-pH can increase exosome uptake efficiency. It has been proved that membrane composition changed by low-pH influence exosome uptake by homologous cells, but it was not explored under hypoxia. It remains unknown that which of exosome uptake methods is more obviously changed by hypoxia

After exosomes or EVs are recognized and bound to recipient cells, they may be internalized by different types of clathrin-independent endocytosis, including macropinocytosis, phagocytosis and endocytosis via caveolae and lipid rafts, or clathrin-dependent endocytosis (Fig. 1) [27, 56, 165, 175]. Exosome uptake is energy-dependent [175–177], and hypoxia indicates that cells undergo energy reprogramming. Dynamin2 is a GTPase required for clathrin-mediated endocytosis [165] whose promoter has HIF-binding sites that are reduced in epithelial ovarian cancer cells under hypoxia by HIF-1α and can reciprocally regulate HIF-1α via an iron-dependent mechanism [178]. The endocytic adaptor Eps15 also affects clathrin-mediated endocytosis [165], and PHD3 controls EGFR internalization by affecting the recruitment of Eps15 and epsin1 to EGFR [179]; whether exosomes take part in these processes is unknown. Caveolin-1 is important for caveolin-dependent endocytosis. Two respective reports showed that caveolin-1 is elevated to enhance the malignant characteristics of different tumor cells under hypoxic conditions [180, 181]. Compared to normoxic conditions, hypoxic conditions reduced macropinocytosis by ~ 50% in AT1 cells, whereas this process was largely O_2_-independent in Walker-256 cells [182]. The researchers also reported that extracellular acidosis, which is tightly associated with hypoxia, exerts cell line-specific influences on macropinocytosis, clathrin-mediated endocytosis and cholesterol-dependent endocytosis [182]. EV uptake through phagocytosis is associated with PI3Ks [165], and it has been reported by many different researchers that hypoxia regulates pathways involving PI3Ks in various physiological processes [183–185]. Phosphatidylserine, which was discussed above, is also involved in both phagocytic and macropinocytic uptake of EVs [165], but we know little about its interaction with hypoxia in exosome uptake. Hypoxia also increases exosome uptake by lipid raft-dependent endocytosis [150]. Interestingly, exosomes can specifically bind to and remain at the plasma membrane of follicular dendritic cells, providing MHC class II molecules [186]. Two recent studies showed that EV content release also depends on energy, acidic pH and proteins present on the surface of both EVs and PM-derived membranes [176, 177]. Whether this exosomal behavior is similar in cancer cells and whether hypoxia participates in this behavior remain unknown.

## Exosome-mediated biofunctions in cancer under hypoxia

**Fig. 2:**
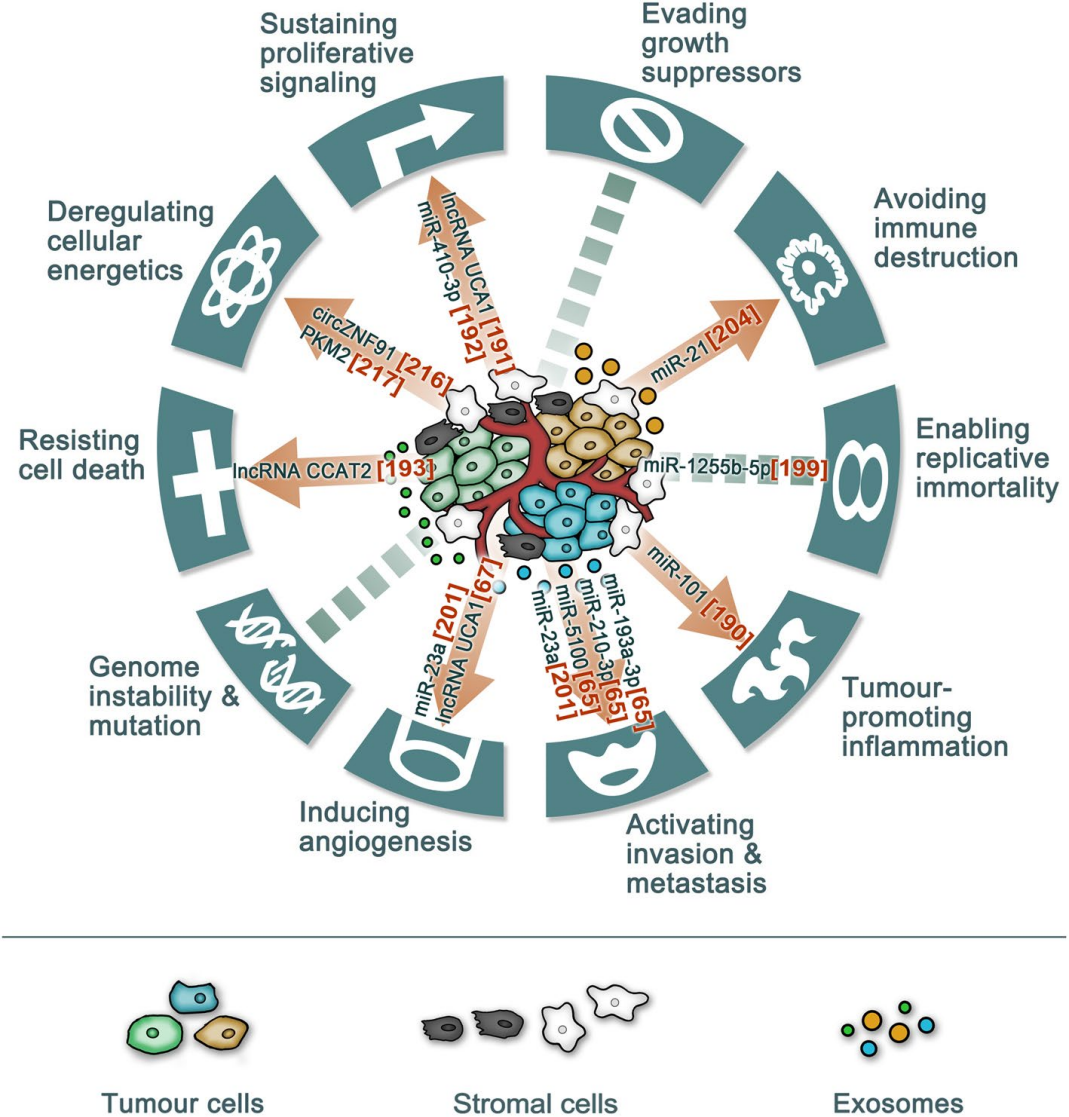
The heterogeneities of exosomes and their biofunction in the hypoxic TME. Different colors for cells represent different kinds and status (such as hypoxia) of cells in tumour microenvironment, indicating tumour heterogeneities. Different colors for exosomes represent heterogeneities in exosomal cargoes and cellular origins. Similar colors between cells and exosomes represent heterogeneities in exosomal origin. Different diameters represent heterogeneities in exosomal size. The outer circle of the picture represents the hallmarks of cancer. Red arrowhead represents that hypoxic tumour-derived exosomes influence corresponding cancer hallmarks and these arrowheads indicate heterogeneities in biofunctions medicated by exosomes. Blue dotted line represents it is too early to draw a conclusion that corresponding hallmarks are affected by hypoxic tumour-derived exosomes [190]

Hanahan and Weinberg, well-known leaders in cancer research, summarized the acquired capabilities of cancer cells [73, 187], and with the advancement of cancer research, other hallmarks and oncological factors (the microbiota, obesity and autophagy) have been found [72, 74, 75, 188, 189]. Exosomes from cancer cells or cancer-related cells can mediate many biofunctions, and hypoxia strengthens these biofunctions (Fig. 2). Here, we provide minimal representative but not exhaustive examples of some aspects.

### Proliferation, apoptosis and growth suppression

Self-sufficient growth signals, downregulated antigrowth factors and decreased apoptosis cause cells to propagate rapidly. Tumor proliferation is usually promoted by HypoExo cargoes, such as exosomal lncRNA UCA1 from hypoxic bladder cancer cells [191] and exosomal miR-410-3p from hypoxic CRC cells [192], and apoptosis of tumor cells is usually inhibited by exosomes under hypoxia. For example, hypoxia-induced apoptosis can be inhibited by exosomal lncRNA CCAT2 released from glioma cells via increased expression of the antiapoptotic regulator Bcl-2 and downregulation of the proapoptotic factor Bax in recipient cells [193]. However, considering the antiproliferative effects of growth-suppressing factors, it is reasonable to speculate that exosomes released from hypoxic cancer cells do not transmit these adverse factors to their neighboring cells. Regardless, cancer cells can secrete exosomal TGF-b, a well-known anti-growth factor, to achieve immunosuppression under hypoxia [194], which may be related to exosome-specific uptake. In addition, the growth-suppressing factors TGF-β and liver kinase B1 (LKB1) in cancer cells can increase exosome release to promote the proliferation and migration of target cells [195, 196], although the effects of hypoxia on this process are unknown. These results highlight the complexities in regulating the network of pro- and anti-growth in tumors and exosomes under hypoxia, which tends to induce protumor conditions.

### Immortal replication

Limitless replicative potential provides an endless source for cancer cell proliferation and is tightly associated with telomeres and senescence [73, 187]. Telomeric repeat-containing RNA (TERRA) can protect short telomeres to maintain cell mitosis [197], and TERRA exists in exosomes released from a human lymphoblastoid cell line and has a proinflammatory function [198]. Another study more focused on HypoExos and immortal replication showed that CRC-derived exosomal miR-1255b-5p can target human telomerase reverse transcriptase to suppress tumors and that hypoxia decreases exosomal miR-1255b-5p [199]. However, it is too early to draw a conclusion about the role of exosomes in immortal replication under hypoxia, and more studies are needed.

### Angiogenesis, invasion and metastasis

Angiogenesis is a process that addresses nutrient and oxygen needs and the requirement of evacuating metabolic wastes and carbon dioxide [73], while invasion and metastasis are the main causes of cancer-related death [76].

Important studies have proven that hypoxia induces angiogenesis, and they are summarized in some reviews [200]. Recently, an increasing number of studies have assessed the role of exosomes in hypoxia-induced tumor angiogenesis. For example, HypoExos derived from highly malignant glioblastoma multiforme cells induce angiogenesis by stimulating cytokine and growth factor secretion by endothelial cells, thereby promoting pericyte migration [57]; HypoExo exosomal lncRNA UCA1 in pancreatic cancer promotes angiogenesis via the miR-96-5p/AMOTL2/ERK1/2 axis [67]; HypoExo miR-23a increases angiogenesis by targeting prolyl hydroxylase in lung cancer [201]. There are many additional examples. Considering the clinical applications of antiangiogenic therapy, targeting these proangiogenic exosomes may provide a new avenue for solid tumor treatment.

The function of HypoExos in promoting invasion and metastasis is definite, as proven in studies of HypoExos from RCR [116, 192], bladder cancer [191], gastric cancer [21], esophageal squamous cell carcinoma [202] and lung cancer [105]. However, tumor metastasis is a multistep process known as the invasion−metastasis cascade [76, 203]. Thus, we should further explore the role of exosomes from hypoxic tumors in each step from in primary tumor cell development to metastasis formation. For example, exosomal miR-193a-3p, miR-210-3p and miR-5100 from hypoxic BMSCs activate STAT3 signaling-induced EMT [65], which may increase cancer cell motility, invasiveness, and ability to degrade extracellular matrix (ECM) [76]; hypoxic lung cancer-secreted exosomal miR-23a increases vascular permeability by inhibiting tight junction protein ZO-1 [201], which may facilitate the transendothelial migration of tumor cells. HypoExos form PMNs at distant metastatic sites [81, 82], which is convenient for CTC immigration. The roles of HypoExos in other metastasis steps, such as the effects of HypoExos on tumor cells entering and exiting dormancy, the communication between CTCs or circulating tumor cell clusters and other circulating components (monocytes, NK cells, neutrophils and platelets) and the signaling network promoting the initiation of metastatic colonization and metastatic evolution, remain unexplored. More studies are expected to inform these ideas.

### Immune response

Since the success of checkpoint blockade (such as agents targeting PD-1 or PD- L1), immunotherapy has become a well-established treatment modality for cancer. Reversing immune suppression and promoting immune activity are two major strategies of immunotherapy, while signaling pathways and metabolic reprogramming are two major regulators of the immune response mainly induced by immune-related ligand−receptor binding. Here, we first focus on signaling pathways and take PD-1/PD-L1 as an example for ligands and receptors. Regarding signaling suppression, the surface expression of ligands or receptors is the first immune response regulator, and HypoExos can change their expression. HypoExo miR-21 from oral squamous cell carcinoma (OSCC) increases the PD-L1 expression of MDSCs, thus decreasing the antitumor ability of γδ T cells [204]; lung cancer-derived EVs from intermittent hypoxia increase the PD-L1 expression of macrophages, thus aggravating the immunosuppressive status in macrophages [205]. Exosomal circ-0001068 can induce PD-1 expression in T cells [206], but whether HypoExos have similar functions is unknown. In addition, exosome cargoes may be immune ligands. For example, PD-L1 carried by exosomes can directly interact with T cells to suppress antitumor ability, which has been proven with exosomes from melanoma [207], breast cancer [208], prostate cancer [209], head and neck cancer [210], pancreatic cancer [211] and gastric cancer [212]. Therefore, exploration of the effects of effects of hypoxia on loading of PD-L1 onto exosomes is needed. Moreover, hypoxic tumor exosomes may directly influence factors downstream of ligand−receptor pathways in a way that enables them to bypass the ligands or receptors to cause an immune response.

### Metabolism reprogramming

Cancer cells undergo metabolic reprogramming even under normoxia (for example, the Warburg effect [148]), and cell metabolism under hypoxia also changes into something barely recognizable. Metabolism has great impacts on cancer biology, so it is essential to explore the role of exosomes in metabolism in the hypoxic TME. As mentioned above, the effects of hypoxia and exosomes interact. PKM2, a glycolytic pyruvate kinase isoenzyme increased by hypoxia [44], can increase exosome secretion [45], which is an example of metabolic reprogramming under hypoxia that affects the biogenesis of exosomes, and hypoxia results in metabolite changes that may influence the loading of cargoes into exosomes. In the next section, we will focus on exosomal impacts on metabolism in a hypoxic TME. The composition of exosomes can reflect the hypoxic status of tumor cells [57], and this can be used for tracking metabolic reprogramming [48]. Moreover, both normoxic exosomes and HypoExos can reprogram the metabolism of receptor cells, including infiltrating immune cells. Exosomes from normoxic CAFs can inhibit mitochondrial oxidative phosphorylation, thereby increasing glycolysis and glutamine-dependent reductive carboxylation in cancer cells, which is similar to hypoxia-induced metabolic alterations [213], and exosomes from tumor cells can activate hepatic stellate cells to secrete IL-6, which then regulates the lactate metabolism of hypoxic tumor cells [214]. HypoExos from tumor cells can enhance oxidative phosphorylation in infiltrating monocytes−macrophages via transfer of let-7a miRNA, resulting in suppression of the insulin-Akt-mTOR signaling pathway [215], and HypoExos (for example, HypoExo circZNF91 from pancreatic cancer cells [216] and HypoExo PKM2 from NSCLC [217]) can promote glycolysis in receptor cells, resulting in treatment resistance. Exosomes may supply nutrients for hypoxic tumor cells. Zhao et al. conducted an intraexosomal metabolomics study and concluded that CAF-derived exosomes may provide various metabolites, including amino acids, lipids, and TCA cycle intermediates, that are avidly utilized by cancer cells under nutrient deprivation or nutrient stress conditions [213]. In addition to affecting nutrient supply, these exosomal metabolites have great possibilities to influence target cells, especially immune cells. The hypoxia–lactate axis is an important regulator of tumor immunity [218]. Therefore, it is possible that absorption of exosomal lactate can greatly assault tumor immune cells; unfortunately, this idea remains to be confirmed.

### Therapeutic resistance

Similar to infectious diseases, cancers often become resistant to various therapies, from traditional chemotherapy and radiotherapy to targeted therapy and immunotherapy. Even more discouraging is the fact that therapeutic resistance also occurs with drug combinations. With the goal of utilizing them for better tumor treatment, the mechanisms underlying therapeutic resistance have received constant attention. Dysfunctional neovasculature creates a hypoxic TME and decreases the effective exposure of a tumor to drugs, linking hypoxia to therapeutic resistance. Moreover, exposure to hypoxia results in metabolic reprogramming, which also makes tumor cells resistant to various therapies. Many previous studies have proven that exosomes can transmit therapeutic resistance from insensitive tumor cells to sensitive cells, culminating in a more malignant tumor or reoccurrence. Thus, the study of exosomes in the hypoxic TME is needed. The aforementioned metabolic reprogramming induced by HypoExos after uptake and neutralization of drugs by metabolites in HypoExos are the main causes of treatment resistance [216, 217]. Another possibility is that HypoExos influence drug resistance through drug efflux or drug sequestration. miR-155 can be used as an example. miR-155 is overexpressed in HypoExos from glioma [219] and hepatocellular carcinoma [19] and hypoxic TAMs [220] and is associated with cisplatin resistance [221]. miR-155 inhibitor-laden exosomes can reverse cisplatin resistance by suppressing drug efflux transporter protein expression in cisplatin-resistant OSCC [221], but whether HypoExo miR-155 can induce drug resistance by upregulating drug efflux is unknown. Single pathways are also utilized by HypoExos to promote drug resistance. For example, HypoExo miR-301a can inhibit TCEAL7 and relieve the suppression of the Wnt/β-catenin pathway, causing radiation resistance [222]. The anti-apoptotic pathway is another example. HypoExo miR-21 from NSCLC can downregulate PTEN, an important tumor suppressor related to apoptosis, to induce cisplatin resistance [223]. There may be additional mechanisms underlying resistance to targeted therapy and immunotherapy. First, we discussed the role of HypoExos in immune checkpoint blockade by using PD-1/PD-L1 as an example. Moreover, exosomes, such as Her-2+ exosomes [224], can function as competitors with tumor cells, decreasing the targeting ability of antibody-based drugs, and this idea is worth exploring under hypoxic TME conditions.

## Exosomes in cell-to-cell communications in THE hypoxic TME

Hypoxia, along with other selective pressures, promotes the convergent evolution and diversification of both malignant and nonmalignant cell compartments, including cancer-associated fibroblasts (CAFs), tumor-infiltrating lymphocytes (TILs), tumor-associated macrophages (TAMs) and dendritic cells (DCs), of the TME, resulting in spatiotemporal evolution of intratumoral heterogeneity (ITH) [225]. For example, in hypoxia, the fact that oxygen supply in the tumor decreases with the distance from the disordered neovasculature reflects spatial ITH, and the fact that a hypoxic zone arises with tumor progression reflects temporal ITH. Because of the paramount impacts of ITH on tumor progression and response to treatment, investigating the communication between the compartments of the TME, monitoring dynamic ITH and targeting ITH are important. In the next section, the roles of exosomes in the communication between the compartments in the hypoxic TME are discussed.

Exosomes link malignant and nonmalignant compartments of the TME not only under normoxia but also under hypoxia. Exosomes from hypoxic CAFs can promote breast cancer cell stemness through circHIF1A [226], and exosomes from hypoxic NSCLC cells can reprogram CAFs to form an acidic microenvironment causing NSCLC cell proliferation and cisplatin resistance [217]; exosomes from hypoxic TAMs can promote chemoresistance in ovarian cancer cells through the miR-223/PTEN-PI3K/AKT pathway [227]. In addition, exosomes from different tumor cells under hypoxia [215, 228, 229] can promote TAM M2 polarization through different miRNAs, and exosomes from hypoxic CRC tumor cells can educate distant Kupffer cells (macrophages) to form PMNs for liver-tropic metastasis [81]. Exosomes from hypoxic granulocytic MDSCs can promote the stemness of CRC cells through S100A9 [15], and exosomes from hypoxic glioma cells can induce stronger MDSC expansion and activation through the miRNA-10a/Rora, miRNA-21/PTEN, miRNA-29a/Hbp1 and miRNA-92a/Prkar1a pathways [230, 231]. Cargoes in DC-derived exosomes are changed by hypoxia [232], but the interaction between DCs and tumor cells in the hypoxic TME remains largely unexplored and needs more attention. Interestingly, exosomes from hypoxic NK cells have stronger instead of decreased antitumor ability [233], and MVs (not exosomes) from tumor cells can weaken the antitumor effects of NK cells through TGF-β and miR23a [234]. However, more details need to be elucidated. Exosomes from hypoxic breast cancer cells suppress T cell proliferation via TGF-β [194] while studies about the impacts of exosomes from hypoxic lymphocytes on tumor cells are lacking at present. The above studies highlight the interactions of tumor cells and noncancerous cell compartments in the hypoxic TME, but more studies are needed to produce evidence-based support. In addition, studies have proven that noncancerous cell compartments can affect other compartments during tumor progression (for example, exosomes from hypoxic DCs [235] or TAMs [236] can affect other immune cells), but studies of this phenomenon under hypoxic circumstances are rare and are warranted.

## Clinical application

Precision oncology and liquid biopsy applications have advanced with the development of precision medicine. Traditional tissue biopsy samples taken from a single or few spatial areas may be limited and unable to reflect the tumor state of the whole body. Liquid biopsy strategies that assess factors including circulating tumor DNA (ctDNA) [237, 238], EVs or exosomes [239], circulating tumor cells (CTCs) [238] and other biochemical substances seem to have the ability to overcome these disadvantages. Exosomes are unique among these biochemical factors because exosomes contain not only DNAs but also various proteins, RNAs, glycoconjugates and lipids, showing more potential clinical utility, and are more accessible due to being detectable in almost all bodily fluids, which makes them ideal biomarkers for monitoring dynamic ITH to provide useful clinical information for diagnosis and prognosis prediction; such characteristics allow dynamic adjustment of treatment for a better outcome and minimize toxicity and side effects. For example, imaging techniques can only detect cancer when it is morphologically visible, and by that time, the cancer is relatively advanced and may even have metastasized. Circulating exosomes are useful for early diagnosis, allowing timely clinical intervention. Monitoring dynamic ITH with exosomes may help to address possible adverse events promptly. Taking advantage of accessible circulating exosomes may avoid unexpected complications induced by contrast medium or invasive tissue biopsy. Using exosomes to predict treatment response avoids harm caused by the drugs in patients with drug insensitivity. This field is experiencing exciting developments. Many clinical trials of exosomes as a tool for diagnosis or prognosis assessment are being conducted (Table 1). Exosome biomarkers related to hypoxia reflecting not only the hypoxic status of tumors but also tumor aggression and prognosis are also appealing. For example, hypoxia-regulated mRNAs and proteins (such as MMPs, IL-8, PDGFs, caveolin 1, and lysyl oxidase) are enriched in hypoxic glioma cells, and patients with high levels of some of these markers tend to have worse survival [57]. Another study focusing on miRNAs suggested that low levels of exosomal miR-486-5p and miR-181a-5p and high levels of exosomal miR-30d-5p in plasma are related to hypoxia and high-risk rectal cancer [64]. All of the above are potential HypoExo biomarkers. However, HypoExo biomarkers in cancer cells lack validation in large-scale clinical trials.

Strategies to target and remove circulating oncogenic biohazards have been explored, and the same therapies have also been applied to target specific exosomal biomarkers [240]. A recent study took advantage of mesoporous silica nanoparticles (MSNs) with EGFR-targeting aptamers; these nanoparticles interacted with circulating cancer-derived EGFR^+^ exosomes and eliminated these exosomes, causing their entry into the small intestine, which reduced the formation of metastasis [241]. HypoExo biomarkers may provide more target sites. Studies in these fields remain deficient, but more are anticipated in the future. Another clinical application of exosomes is to take advantage of their natural antitumor properties. Exosomes from immune cells, even tumor cells, have antitumor abilities (for more details, please see the review by Moller and Lobb) [13]. Thus, how to exploit or target hypoxia to increase antitumor cargoes in exosomes is worth exploring. For example, exosomes from hypoxic NK cells have a strong antitumor ability [233]. Additional studies on this topic are needed to establish the feasibility of the in-human use and large-scale production of antitumor exosomes. In addition to the use of exosomes from cells, artificial engineering, such as electroporation, reagent transfection, sonication, freeze–thaw cycles or saponin methods, is another method of loading functional cargoes or drugs into exosomes [13]. Hypoxia may affect the efficiency of therapy by influencing the suitability of exosomal cargoes as scaffolds for fusing functional molecules and other agents. Moreover, exosomes in the hypoxic TME show specific uptake, which may provide an avenue for specific targeting of malignant cells. Other problems include how to load drugs into exosomes rapidly and effectively, how to enhance the stability of exosomes to ensure a longer half-life and how to improve specific targeting. Reports on these research topics are promising. Lathwa et al. extended the lifetime of exosomes to 12 h, compared with 3 h for natural exosomes, by applying biocompatible photomediated atom transfer radical polymerization (ATRP) [242]. Xiaojuan Zhang developed a new special and effective functional protein- and ribonucleoprotein-loading strategy by designing EVs that coencapsulate vesicular stomatitis virus G protein (VSV-G) with bioactive macromolecules via split GFP complementation [243].

## Conclusions and future

With tumor expansion, hypoxia occurs. To survive in such a microenvironment, tumor cells take various actions and release exosomes to transmit signals to other cells to trigger cancer-promoting effects (such as signals to induce angiogenesis) or defensive effects (such as signals to induce invasion and metastasis). Given the incomplete understanding of hypoxia and exosomes, a perfect framework for understanding exosomes in hypoxia cannot be established. However, we still conclude the following:

1. Hypoxia often, though not always, increases the secretion of exosomes in various tumor cells through direct (cargo sorting, transport of MVBs and fusion with the plasma membrane) and indirect (metabolic reprogramming, induction of an acidic microenvironment, and effects on calcium and other regulatory molecules) methods, but a more detailed mechanism is needed.
2. Hypoxia impacts exosome heterogeneity in terms of size, cargo and cellular origin to change biofunctions.
3. Exosome size alterations may reflect and contribute to ITH evolution, but it is too early to draw a valid conclusion due to the lack of evidence regarding how hypoxia affects exosome size.
4. Exosome cargoes are different in hypoxic and normoxic TMEs. Hypoxia may influence the biosynthesis, metabolic degradation and postsynthesis modification of cargoes and the efficiency of special cargo-sorting mechanisms.
5. The hypoxic microenvironment may facilitate exosome transport due to exosomal acidophily. Moreover, hypoxia may also influence exosome target cell recognition and exosome internalization by changing recognition molecules and various internalization pathways via auxiliary or indirect methods, such as affecting energy supply and inducing low pH.
6. Exosomes in the hypoxic TME usually play a pro-tumor role and sometimes have antitumor abilities. Signaling pathway mediation and metabolic reprogramming of receptor cells are two major regulatory methods used by exosomes to play biological functions in the hypoxic TME. Competitive interaction with drugs is another nonnegligible role of exosomes in therapeutic resistance.
7. Hypoxia is one of the main causes of ITH evolution, and exosomes in the TME can be used to investigate the communication between the compartments of the TME, monitor dynamic ITH and target ITH. Moreover, exosomes are a universal communication mechanism used between malignant−malignant compartment, malignant−nonmalignant compartment, and nonmalignant−nonmalignant compartment interactions in the hypoxic TME.
8. Exosomes are ideal biomarkers for monitoring dynamic ITH to provide useful clinical information for diagnosis and prognostication, allowing treatment alteration for a better outcome and minimizing toxicity and side effects. In addition, removing and targeting protumor exosomes, utilizing antitumor exosomes and using exosomes engineered to carry specific cargoes or drugs for cancer therapy are other potential clinical applications. Integrating these strategies with strategies related to hypoxia may provide new avenues for cancer treatment, for example, strategies to specifically target malignant cells.

The future of this field is worthy of discussion. First, rapid, reliable and inexpensive exosome isolation techniques that do not induce contamination are urgently needed. Fortunately, some advances have been made. In addition to the polyethylene glycol (PEG)-based precipitation, phosphatidylserine affinity capture, size-exclusion chromatography and membrane affinity processes, and asymmetric flow field-flow fractionation methods in existence, Chen et al. developed the EXODUS method, a new ultrafiltration strategy that achieves clog-free and ultrafast purification of exosomes with improved speed, yield and purity [244]. However, as methods evolve, new problems occur. For example, analysis of exosomes captured by an advanced method showed that argonaute1–4, glycolytic enzymes and cytoskeletal proteins were not detected in the exosomes [103], which contradicts previous studies. Whether the previous results were affected by imperfect isolation techniques or whether the inconsistent observations reflect exosome heterogeneity due to different cell origins and states remains unclear. Although there are no doubts about exosomes containing miRNAs, studies have shown that there is far less than one molecule of a given miRNA per exosome [245], increasing the ambiguity surrounding exosomes and indicating that explorations of exosome heterogeneity at the single-vesicle level are needed. Fortunately, some single-vesicle protocols, including single-vesicle tracking, will likely make these types of studies possible in the future (details in ref. [246]). Second, tracking exosomes in vitro and in vivo can provide us with more knowledge. Although different tracking methods have been explored, including intravital imaging with the help of anesthesia [247], magnetic particle imaging (MPI) tracking of superparamagnetic iron oxide (SPIO) labels [18], tracking of fluorescence with a pH-sensitive dye (pH-sensitive tetraspanin TSPAN [248], CD63-pHluorin [249] or mutant CD63-pHluorin [250]) and 3D tracking [251], they are not sufficiently mature for use in massive and broad applications in vivo. Single parent cell-single exosome or EV-single target cell tracking is the ideal method, and we promising studies are awaited. Currently, the development of strategies using exosomes to reflect malignant and nonmalignant cell compartments is lagging behind the development of ITH. In recent years, single-cell sequencing has led to substantial developments in ITH, and single-cell atlases have revealed distinctions between cancer cell subpopulations, creating challenges and opportunities. For instance, analysis of single-cell RNA has revealed intrinsic subpopulations underlying prostate tumor subtypes (16 clusters for epithelial cells, 7 clusters for monocytic cells and 7 clusters for T cells), and some of these cell subpopulations were found to be important [252]. Single-cell RNA sequencing of lung cancer cells collected at different times, including before initiation of systemic targeted therapy (TKI naive [TN]), at the occurrence of residual disease (RD) and at the occurrence of drug resistance, revealed treatment-induced transformation of the primordial tumor cell state [253], but single-cell RNA sequencing integrated with spatial transcriptomics analysis revealed that different tumor cell subpopulations have spatially restricted enrichments as well as distinct coenrichments with other cell types [254]. Therefore, we must consider how different cell subpopulations communicate and coordinate with each other. The roles and changes of exosomes from different subpopulations are more complex in these networks at different times and in different spaces, which may contribute to the reconstruction of a precise TME architecture. In addition to these exciting challenges, single-cell sequencing also provides a new analysis strategy. For example, Chen et al. found enrichment of exosome-associated genes and a general lack of upregulation of androgen receptor (AR) signature genes, suggesting that accumulation of KLK3 levels in prostate cancer CD8+ T cells was mediated by tumor-derived EVs. Further experiments confirmed this finding [252]. In addition, we can also use powerful single-cell sequences to explore the role of hypoxia in tumors [255]. For example, through single-cell analysis, researchers have found that colon cancer TAMs and some prostate cancer epithelial cells have hypoxic signatures [252, 256]. In the future, with development of single-cell transcriptomics and spatial transcriptomics strategies, the challenges will increase, as will the opportunities.

## Data Availability

Not applicable.

## Abbreviations

AGO2: Argonaute 2
ANGPLT4: Axosome-derived protein of angiopoietin-like 4
AR: Androgen receptor
ATRP: Atom transfer radical polymerization
BMSCs: Bone marrow-derived mesenchymal stem cells
BOS: Bronchiolitis obliterans syndrome
Col-V: Collagen V
CRC: Colorectal cancer
CTCs: Circulating tumour cells
ctDNA: Circulating tumour DNA
ECM: Extracellular matrix
EGFR: Epidermal growth factor receptor
ESEs: Early-sorting endosomes
EVs: Extracellular vesicles
G-MDSCs: Granulocytic myeloid-derived suppressor cells
hnRNPA2B1: Heterogeneous nuclear ribonucleoprotein A2B1
HREs: Hypoxia response elements
HSPG: Heparan sulfate proteoglycans
HUVECs: Human umbilical cord endothelial cells
ICAMs: Intercellular adhesion molecules
ILVs: Intraluminal vesicles
Kα1T: K-α-1-tubulin
LKB1: Liver kinase B1
lncRNAs: Long non-coding RNAs
LSEs: Late-sorting endosomes
LTxR: Lung transplant recipients
miRISC: miRNA­induced silencing complex
miRNAs: microRNA
MPI: Magnetic particle imaging
MVBs: Multivesicular bodies
MVEs: Multivesicular endosomes
MVs: Microvesicles
NSCLC: Non-small cell lung cancer
PA: Phosphatidic acid
PC: Phosphatidylcholine
PE: Phosphatidylethanolamine
PEG: Polyethylene glycol
PI: Phosphatidylinositol
PKM2: Pyruvate kinase M2
PS: Phosphatidylserine
PTMs: Post-translational modifications
RBM4: RNA binding motif protein 4
RBPs: RNA-binding proteins
rHREs: Reverse hypoxia response elements
SNAP: Synaptosome-associated protein
snRNAs: Small nuclear RNAs
SPIO: Superparamagnetic iron oxide
SV2: Synaptic vesicle protein 2
SYNCRIP: Synaptotagmin-binding cytoplasmic RNA-interacting protein
S1P: Sphingosine1-Phosphate
TERRA: Telomeric repeat-containing RNA
TME: Tumour microenvironment
tRNAs: Transfer RNAs
VSV-G: vesicular stomatitis virus G protein
YBX1: Y-box-binding protein 1
